# The effect of sad facial expressions on weight judgment

**DOI:** 10.3389/fpsyg.2015.00417

**Published:** 2015-04-10

**Authors:** Trent D. Weston, Norah C. Hass, Seung-Lark Lim

**Affiliations:** Department of Psychology, University of Missouri-Kansas City, Kansas City, MOUSA

**Keywords:** emotions, facial perception, facial expressions, obesity, body weight

## Abstract

Although the body weight evaluation (e.g., normal or overweight) of others relies on perceptual impressions, it also can be influenced by other psychosocial factors. In this study, we explored the effect of task-irrelevant emotional facial expressions on judgments of body weight and the relationship between emotion-induced weight judgment bias and other psychosocial variables including attitudes toward obese persons. Forty-four participants were asked to quickly make binary body weight decisions for 960 randomized sad and neutral faces of varying weight levels presented on a computer screen. The results showed that sad facial expressions systematically decreased the decision threshold of overweight judgments for male faces. This perceptual decision bias by emotional expressions was positively correlated with the belief that being overweight is not under the control of obese persons. Our results provide experimental evidence that task-irrelevant emotional expressions can systematically change the decision threshold for weight judgments, demonstrating that sad expressions can make faces appear more overweight than they would otherwise be judged.

## Introduction

Obesity is more than just a health issue. Being overweight or obese can have significant psychosocial implications for the individual (Puhl and Heuer, 2009). For example, overweight or obese persons are more likely to experience discrimination in the workplace, such as lower job performance ratings (O’Brien et al., 2008; Nieminen et al., 2013) and lower wages (Baum and Ford, 2004). Additionally, obesity has been shown to impact interpersonal experiences. Overweight or obese persons are more likely to be perceived as less attractive, less trustworthy, or less healthy (Hume and Montgomerie, 2001; Miller and Lundgren, 2010; Coetzee et al., 2011). The bias against obesity has grown into a culture of negative social evaluations and consequences for overweight individuals. Furthermore, the stigma of being overweight or obese is associated with negative psychological functioning such as depression, poor self-esteem and stress (Wadden and Stunkard, 1987; Friedman et al., 2005; Major et al., 2012). Thus, coping with the social effects of being overweight or obese can have enduring cognitive, physical, and emotional consequences on the individual.

Although the stigma of being deemed overweight or obese can have significant negative consequences, perceptual judgments of another person’s body mass (e.g., normal vs. overweight) are largely subjective and often biased through many psychosocial factors. The weight, gender, eating problems, body weight preoccupation, depression, self-esteem, or emotional instability of an individual can influence his or her body size perception of another person (McCabe et al., 2006; Sand et al., 2011). Furthermore, body weight perception also can be influenced by contextual factors. For instance, observing the meal-size an individual consumed can systematically influence subsequent weight judgments of the observer on an identical eater (Vartanian et al., 2008). Whereas body mass index (BMI; kg/m^2^) is widely utilized as an objective measurement of body weight status based on height and weight, judgments of weight more often rely on subjective, perceptual impressions that can be easily biased by psychosocial factors not directly relevant to objective height or weight. Due to the pervasive influences of the mass media that transmit a distorted standard of healthy body images (Murray et al., 1996), subjective judgments of weight status (normal or overweight) on others as well as ourself may not necessarily correspond to actual medical judgments. Body image is easily influenced by social context such as family, peers, and mass media messages (Hogan and Strasburger, 2008). Many individuals in normal or underweight BMI status are afraid of being fat and express a strong desire to lose body weight (Robison et al., 1993). During everyday social interactions, we easily and quickly judge other’s weight status (e.g., thin, normal, or fat) relying on subjective perceptual impressions without objective information such as a BMI score that is necessary for the medical classification of obesity. If we perceive someone as obese, then our subsequent interactions with him or her can be influenced by stereotype or social stigma related to obesity.

Body weight judgments can be made as quickly and simply as by viewing only the face of another individual (Coetzee et al., 2010; Schneider et al., 2012, 2013). Facial judgments in general play a crucial role in social development and functioning. In everyday social interactions, information such as age, ethnicity, mood, intelligence, and personality are often automatically guessed from only brief assessments of the face (Todorov et al., 2013). Body weight is also a salient characteristic that can be quickly retrieved from facial cues. However, all of these characteristic judgments are subject to bias and influenced by various psychosocial variables. In particular, emotional expressions can strongly impact judgments on other individuals in social interactions. For example, emotional expressions have been shown to influence age judgments, specifically resulting in faces with positive expression being significantly underestimated for their age (Voelkle et al., 2012). Emotional expressions have also been shown to influence judgments on trustworthiness and approachability (Willis et al., 2011). Furthermore, previous studies suggest a potential link between the cognitive processing of facial expressions and eating behaviors. For example, facial emotion recognition or attentional processing of facial expressions can be implicated in individuals with high levels of eating psychopathology (Ridout et al., 2012) or obesity (Cserjesi et al., 2011). Specifically, those with high levels of eating psychopathology were more likely to erroneously recognize emotional expressions of facial stimuli (Ridout et al., 2012). Participants with anorexia nervosa demonstrated difficulties in being attentive to positive facial expressions, whereas participants with obesity showed difficulties in being attentive to negative facial expressions (Cserjesi et al., 2011). Also, observing negative and positive facial expressions of others while eating food modulates the desire to consume food products (Barthomeuf et al., 2009). However, it is not known yet whether facial emotional expressions influence weight judgments.

Given the significant impact that being judged as overweight or obese can have on one’s life, it is important to better understand the decision-making mechanisms behind such psychological judgments of body weight. Identifying psychological variables that are entirely irrelevant to weight and height but that systematically modulate subjective judgment may be useful in understanding our perceptual judgment of another’s body weight status, which in turn may prime stereotyped social behaviors related to obesity stigma, is neither objective nor consistent. To our knowledge, it has not been systematically examined how emotional expressions influence subjective perceptional decision-making about body weight. Given prior research showing the impact of emotional expressions on other subjective, psychosocial judgments, we hypothesized that the emotional expressions on faces would systematically bias judgment on body weight. We chose to use sad facial expressions in particular for two reasons. First, obesity is often cormorbid with depression (Preiss et al., 2013; Hoare et al., 2014). Also, previous literature demonstrated a significant association between obesity and attentional processing of negative facial expressions (Cserjesi et al., 2011). Thus, we decided to examine subjective weight judgment in the context of sad facial expressions in the first study of this type.

To test our research hypotheses, we implemented a two-alternative, forced-choice (2AFC) task where individuals were asked to categorize sad and neutral faces of varying weight levels as either “Normal” or “Fat.” This study aimed to identify the effect of a negative emotion on categorical weight decisions through a previously established positive association between social stigma and negative psychological outcomes with overweight status. As such, it was thought that use of the word “Fat” as a categorical label was important, because the possible offensive nature of the word could yield stronger emotional priming (Wadden and Didie, 2003; Volger et al., 2012). A two-alternative forced choice paradigm that has been widely used in psychophysics was chosen to estimate categorical perceptual decision threshold parameters through non-linear psychometric curve fits. We expected that the perceptual decision threshold that determines at what point a face is judged as overweight would be systematically decreased by the presence of task-irrelevant, negative facial affect, resulting in more sensitive (frequent) “fat” decisions for sad faces compared to neutral faces of an equivalent weight due to an association between the concept of being overweight and negative social outcomes (Kring and Gordon, 1998).

We also hypothesized that this effect could be different by the gender of target faces for several reasons as follows. It has been shown that gender modulates affective perception of facial expressions (Kret and De Gelder, 2012). For example, female faces are perceived as more outwardly emotionally expressive than male faces (Kring and Gordon, 1998). Furthermore, sadness is stereotypically linked with females rather than males (Kelly and Hutson-Comeaux, 1999), which might make the sad expression of male faces attentionally salient during the weight judgment of our experiment. Given this, we speculated that male facial images would be more sensitively affected by the task-irrelevant sad emotion for weight judgment in our experiment. Additionally, we hypothesized that the weight judgment bias by sad facial expressions might be positively modulated (i.e., a larger emotional effect on weight judgment) by observers’ own body weight status, depressive symptoms, or explicit attitudes toward obese people, considering established positive associations among body weight, depression, and explicit attitudes about obese people (Hale, 1998; Ip et al., 2013; Preiss et al., 2013; Hoare et al., 2014).

## Materials and Methods

### Participants

Participants consisted of 44 healthy college students with a mean age of 24.0 (SD = 6.7 years; 24 women; 28 Caucasian, 6 African American, 1 Hispanic, 5 Asian, and 4 did not specify) who signed up through the Psych Pool online research participant recruitment system at the University of Missouri – Kansas City (UMKC). Three additional subjects participated, but two did not complete the tasks and one was excluded due to unreliable chance-level responses throughout the entire study. Participants received course credits for completing the experiment, which was conducted individually under controlled, laboratory settings. The study protocol was reviewed and approved by UMKC’s Institutional Review Board, and informed consent was gathered prior to participation. After providing informed consent, body weight and height were measured to calculate BMI (kg/m^2^) by using a digital physician scale (Detecto PD300DHR) in the laboratory. The participants’ BMIs ranged from 17.4 to 44.7 (*M* = 25.6, SD = 5.4). Among the 44 participants, one subject (2.3%) was in underweight status (BMI: 18.5 or lower), 20 (45.5%) were in normal weight status (BMI: 18.5–25), 18 (40.9%) were in overweight status (BMI: 25–30), and 5 (11.4%) were in obese status (BMI: 30 or higher). The participants were not informed of their BMI status during the experiment. After measuring their height and weight, participants were asked to complete self-report questionnaires and experimental tasks.

### Measures

#### Self-Report Questionnaires

Participants completed self-report questionnaires that included the Beck Depression Inventory-II (BDI-II; Beck et al., 1996), and the attitudes toward obese persons (ATOPs) and beliefs about obese persons (BAOPs) scales (Allison et al., 1991). The BDI-II was included to measure the depressive symptoms of participants. In our study, the BDI-II’s Cronbach’s α was 0.88. The ATOP and BAOP scales measured explicit attitudes or beliefs regarding obesity. Higher ATOP scores indicate positive ATOP, while higher BAOP scores indicate stronger beliefs that obesity is not under the obese person’s control. The Cronbach’s α coefficients of the ATOP and BAOP scales were 0.76 and 0.63, respectively.

#### Weight Judgment Task

**FIGURE 1:**
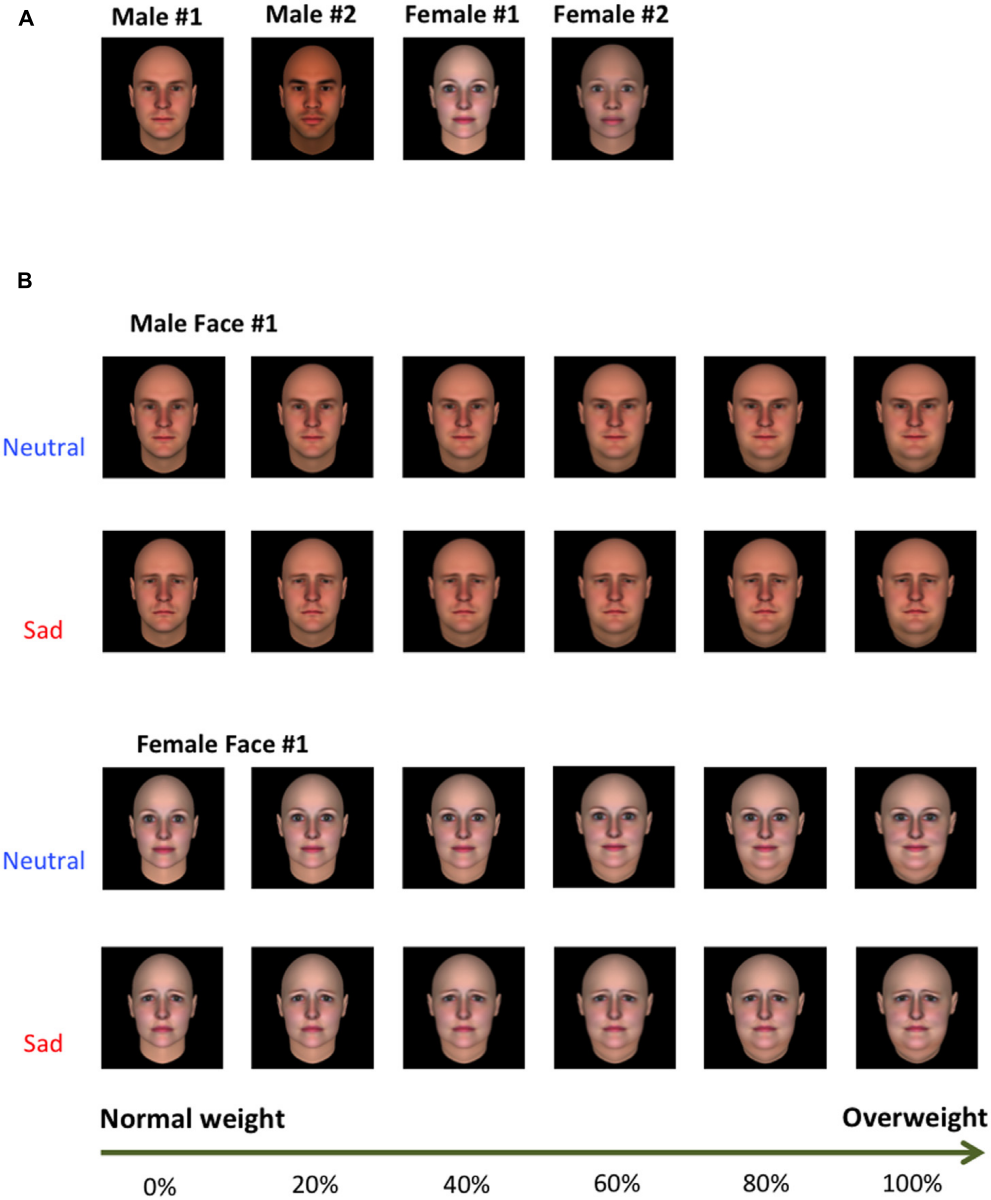
**(A)** Exemplar facial stimuli used for the weight judgment task. A total of four identities (two male identities and two female identities) were used in the main experiment. Normal weight (0%) images are shown. **(B)** Emotional expression and weight of facial stimuli were manipulated by using morphing software. Faces have weight gradients ranging from 0% (normal weight) to 100% (highly overweight) by increments of 20%. Neutral and sad faces are the exact same size and only differ in their emotional expressions.

Participants performed a novel computerized weight judgment task developed to test our research hypotheses. Facial stimuli included four different identities (two male and two female) expressing one of two emotional conditions: neutral or sad (see **Figure 1**). All identities ranged in weight levels from 0% (normal weight) to 100% (extremely obese) by intervals of 20%, such that each of the four identities had six variants of the same size and with the same emotional expression but increasing in their weight. The weight of the faces was scaled in order to create ambiguity when the participants were forced to sort a face as either “normal” (objectively, the 0% face) or “fat” (objectively, the 100% face). Computer-generated facial stimuli were constructed using FaceGen Modeller software (Singular Inversions, Toronto, ON, Canada). The experimental face set was constructed by mixing different races using race-morphing functions of FaceGen Modeller to represent an ambiguous ethnicity. The weight gradients for each facial identity were parametrically manipulated using FantaMorph software (Abrosoft, Lincoln, NE, USA).

To acclimate participants to the structure of our two-alternative forced-choice paradigm, a short practice session (24 trials) with a face set (one male face, one female face) that was unique from the face set of the main task was used. In the weight judgment task, participants were asked to decide whether they would categorize a facial image shown on the computer screen as “Normal” or “Fat” by pressing the keyboard key that corresponded to the respective category. Participants were told to sort the facial stimuli as quickly and as accurately as possible. The experimental schedule of stimulus presentation and behavioral data acquisition were programmed using SuperLab software (Cedrus, San Pedro, CA, USA). A white fixation-cross centered on a black screen appeared first to indicate the location of the stimuli. The top left and right corners of the screen displayed either the category “Fat” or “Normal” in white, 36-point Tahoma font, with the specific category location (left or right) varying randomly between participants. A face (400 by 400 pixels), centered on the screen, was presented for 100 ms after the fixation cross. The participant sorted each face by pressing either “e” or “i” on the keyboard for the left or right category, respectively. After responding, a yellow fixation-cross (duration 500 ms) signified that the participant’s responses were registered. If the participant failed to categorize a face within 2 s, the word “MISS” appeared in red on the screen for a duration of 500 ms. A randomized inter-trial-interval of one to 2 s displayed a blank screen with the fixation-cross before the next trial began. The task was broken into four blocks, each containing the six weight variations of each facial identity in both neutral and sad emotional states, repeated five times (i.e., two male faces/two female faces, two emotional conditions, six weight levels, five times each) for a total of 240 randomized presentations per block. Each block took ∼15 min to complete, making the entire task last slightly over 1 h. We planned a 2 × 2 × 6 (gender of faces by emotion by weight) within-subjects design, and our task was constructed to allow us to observe 40 weight decisions for each condition (cell) of interest in a total of 960 trials. After participants completed the task, they were debriefed and released.

#### Statistical Analysis and Psychometric Curve Fitting

We hypothesized that the emotional expressions of facial stimuli would influence perceptual judgment on the weight of faces by systematically changing the shape of psychometric functions. For each individual, we parameterized psychometric functions and then compared them across different experimental conditions. Relating the proportion of “Fat” responses to the weight levels of the gradually morphed faces, we utilized a psychometric curve-fitting approach that has been successfully employed in previous emotion research (Lim and Pessoa, 2008; Lee et al., 2012; Lim et al., 2014). Following these studies, psychometric curves were fitted by using the Naka-Rushton contrast response model (Albrecht and Hamilton, 1982; Sclar et al., 1990) with an ordinary least square (OLS) criterion.

$$response=\frac{R_{\max}*C^{n}}{C^{n}+{C^{n}}_{50}}+M$$

Here, *response* represents the proportion of “Fat” decisions, *C* is the weight levels of the computer generated face (contrast: 0% ∼ 100% in 20% increments), *C_50_* is the intensity at which the response is half-maximal [also called “threshold” or “point of subjective equality (PSE)”], *n* is the exponent parameter that represents the slope of the function, *R_max_* is the asymptote of the response function, and *M* is the response at the lowest stimulus intensity (0% weight level). Given that the proportion of “Fat” decisions (min 0; max 1) was used, the *R_max_* parameter was constrained to be equal to or less than one and the *M* parameter was constrained to be equal or larger than 0. For each individual’s data, we fitted psychometric curves separately for each type of condition (Male Neutral Faces, Male Sad Faces, Female Neutral Faces, Female Sad Faces). Curve fitting was done with GraphPad Prism software (GraphPad Software, La Jolla, CA, USA).

We hypothesized that our emotional expression manipulation would bias weight perception of faces by changing the subjective perceptual decision threshold (often called PSE) that represents the weight level at which 50% “Fat” decisions occur. This change is often described by the contrast gain model in visual perception research (Reynolds and Chelazzi, 2004; Huang and Dobkins, 2005; Carrasco, 2006) and has been reported in previous studies of affective perception of facial stimuli (Lim and Pessoa, 2008; Lee et al., 2012; Lim et al., 2014). In essence, the contrast gain model predicts that response changes occur at the intermediate levels of stimuli, which is consistent with a horizontal shift of the psychometric curve (*C_50_* parameter shift). In our experimental context, the contrast gain model predicts a decreased weight decision threshold (*C_50_* parameter) for sad faces, resulting in more “Fat” decisions at the intermediate levels of weight in sad faces compared to neutral faces.

## Results

### Weight Judgment Task

To test our research hypotheses, the effect of the task-irrelevant emotional expressions of faces (neutral and sad affect) on the weight perception of facial stimuli was systematically examined by employing repeated-measures ANOVAs and non-linear psychometric curve fitting approaches. For all repeated-measures statistics, we applied Greenhouse–Geisser corrections.

**FIGURE 2:**
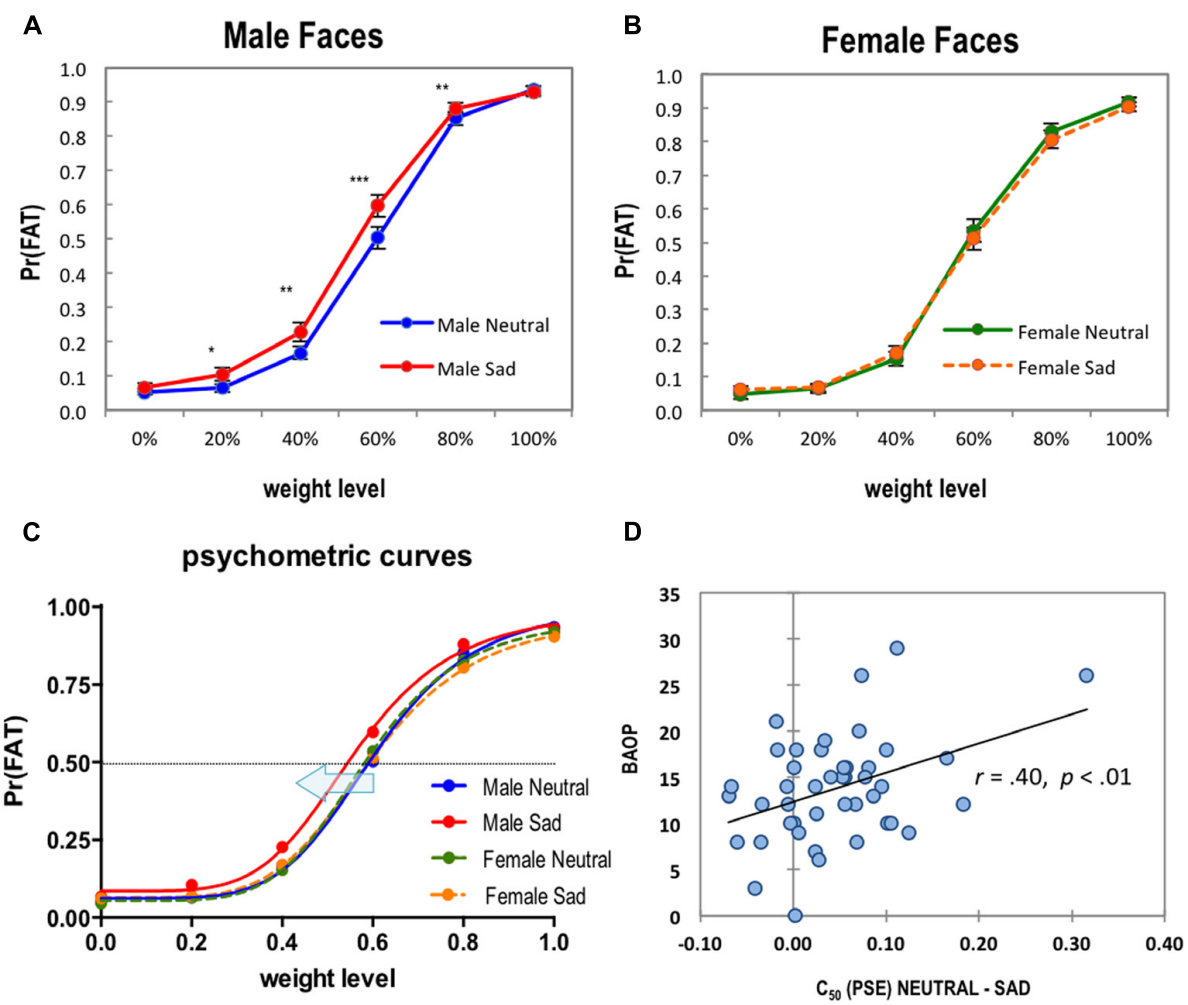
**(A)** Male face data. **(B)** Female face data. Average probability of fat responses as a function of weight levels (0–100% overweight) and emotional expressions. Error bars denote the SE of the mean. ^∗^*p* < 0.01; ^∗∗^*p* < 0.005; ^∗∗∗^*p* < 0.001. **(C)** For the weight judgment data, psychometric curves were fitted by using the Naka-Rushton response function. A leftward-shift of a psychometric curve of Male Sad faces (red line) compared to Male Neutral faces (blue line) was observed. A horizontal dotted line represents the 50% probability of fat decision. **(D)** Scatter plot of the relationship between BAOP (Belief About Obese Persons) scores and C_50_ differences between Male Neutral faces–Male Sad faces. Higher BAOP scores indicate a stronger belief that obesity is not under the obese person’s control. Solid line represents a linear fit.

First, we performed a 2 (GENDER: Male faces, Female faces) by 2 (EMOTION: Neutral, Sad) by 6 (WEIGHT: 0% ∼ 100% in 20% increments) repeated-measures ANOVA on the behavioral data of proportions of fat decisions. Means and SDs are shown in **Table 1**. The ANOVA result on fat decisions revealed a significant three-way interaction effect of GENDER × EMOTION × WEIGHT, *F*(5,215) = 5.37, *p* = 0.001, partial η^2^ = 0.11. We also observed two-way interaction effects of GENDER × EMOTION, *F*(1,43) = 27.31, *p* < 0.001, partial η^2^ = 0.39, and EMOTION × WEIGHT, *F*(5,215) = 5.01, *p* = 0.001, partial η^2^ = 0.10, as well as main effects of GENDER, *F*(1,43) = 7.26, *p* = 0.010, partial η^2^ = 0.14, EMOTION, *F*(1,43) = 5.13, *p* = 0.029, partial η^2^ = 0.11, and WEIGHT, *F*(5,215) = 751.80, *p* < 0.001, partial η^2^ = 0.95. To clarify this three-way interaction effect through simple effect analyses, we performed two-way (EMOTION × WEIGHT) repeated-measures ANOVAs separately for male faces and female faces. Interestingly, the two-way interaction effect of EMOTION × WEIGHT was significant for male faces, *F*(5,215) = 8.18, *p* < 0.001, partial η^2^ = 0.16, but not significant for female faces, *F*(5,215) = 2.28, *p* = 0.070, partial η^2^ = 0.05. This explains the three-way interaction effect we observed – the task-irrelevant emotional expressions of facial stimuli interacted with the morphed weight levels only when judging male faces, but not when judging female faces (see **Figure 2** for graphs). For male faces we observed a significant main effect of EMOTION, *F*(1,43) = 23.30, *p* < 0.001, partial η^2^ = 0.35, as well as a significant main effect of WEIGHT, *F*(5,215) = 691.58, *p* < 0.001, partial η^2^ = 0.94. However, for female faces there was no observable main effect of EMOTION, *F*(1,43) = 0.21, *p* = 0.651, partial η^2^ = 0.01, although we observed a main effect of WEIGHT, *F*(5,215) = 589.05, *p* < 0.001, partial η^2^ = 0.93. Because of the significant interaction effect of EMOTION × WEIGHT for male faces, we performed further simple effect analyses in which the effect of EMOTION was tested for each level of WEIGHT. As shown in **Figure 2A**, participants made a “Fat” categorizing decision more frequently for sad male faces compared to neutral male faces in the 20, 40, 60, and 80% weight levels, *t*(43) = 2.96, *p* = 0.005, *d* = 0.44; *t*(43) = 3.42, *p* = 0.001, *d* = 0.51; *t*(43) = 5.06, *p* < 0.001, *d* = 0.75; *t*(43) = 3.11, *p* = 0.003, *d* = 0.46. As expected, there was no significant difference in any WEIGHT levels by EMOTION in female faces, all *p* > 0.05. Finally, to check whether the sex of participants had any systematic effect on our findings, we performed an exploratory four-way repeated-measures ANOVA that included participants’ sex as an additional between-group factor. However, we could not observe a significant main or three-way interaction effect involving sex (all *p* > 0.05).

**Table 1:** Mean and SD of the proportion of fat decisions.

	Weight level of morphed faces					
Face type	0%	20%	40%	60%	80%	100%
Male neutral	0.052 (0.057)	0.065 (0.079)	0.167 (0.130)	0.503 (0.208)	0.852 (0.131)	0.935 (0.087)
Male sad	0.067 (0.078)	0.105 (0.122)	0.227 (0.178)	0.597 (0.212)	0.880 (0.112)	0.931 (0.093)
Female neutral	0.046 (0.071)	0.064 (0.085)	0.154 (0.127)	0.535 (0.245)	0.828 (0.164)	0.919 (0.092)
Female sad	0.062 (0.080)	0.068 (0.082)	0.170 (0.138)	0.512 (0.225)	0.805 (0.175)	0.904 (0.091)

As stated earlier, we hypothesized that the emotional expressions (neutral or sad) of facial stimuli would influence perceptual judgment on the weight of faces. More specifically, we hypothesized that the perceptual decision threshold that determines binary responses (normal vs. fat) of our two-alternative forced choice task would be modulated by the presence of task-irrelevant negative affect of facial stimuli, resulting in more sensitive (frequent) “Fat” decisions for sad faces compared to neutral faces, even in lower levels of weightiness. The systematic change of the perceptual threshold we hypothesized (i.e., lower decision threshold for sad faces) was tested by comparing psychometric curve fit parameters estimated from each individual. In the Naka-Rushton contrast response model we employed, the C_50_ parameter represents the perceptual threshold or the PSE. The means of C_50_ parameters for male neutral and male sad faces were 0.60 (SE = 0.01) and 0.55 (SE = 0.01), respectively. On the other hand, the means of C_50_ parameters for female neutral and female sad faces were 0.60 (SE = 0.02) and 0.61 (SE = 0.02; see **Table 2** for a full list of parameters). On these C_50_ parameters, we performed a two-way (GENDER × EMOTION) repeated-measures ANOVA. We found a significant interaction effect, *F*(1,43) = 10.73, *p* = 0.002, partial η^2^ = 0.20, and a significant main effect of EMOTION, *F*(1,43) = 5.80, *p* = 0.020, partial η^2^ = 0.12. Subsequent simple effect analyses were conducted separately for male and female faces. As expected, we found a significant difference of C_50_ parameters in male faces, *t*(43) = 4.15, *p* < 0.001, *d* = 0.62, but no difference in female faces, *t*(43) = -1.34, *p* = 0.186, *d* = 0.06. It should be noted that we did not observe any meaningful difference between C_50_ of male neutral faces and C_50_ of female neutral faces, *t*(43) = 0.30, *p* = 0.769, *d* = 0.04, further confirming that the previous significant difference in male faces was indeed due to the decrease of the decision threshold for male sad faces (a leftward horizontal shift of psychometric curve; see the blue arrow in **Figure 2C**), not due to the increase of the decision threshold for male neutral faces. In other words, as the average C_50_ parameters indicate, participants required only 0.56% morphed weightiness to make fat decisions for male sad faces, while they required 0.60% morphed weightiness for male neutral faces. The average decrease across participants was 4.5% (95% CI: 2.3 ∼ 6.6%). For completeness, we performed similar exploratory analyses on other parameters of psychometric curve fits (*M*, *R_max_*, and *n*) although there was no specific hypothesis about these parameters. Not surprisingly, no significant effect was revealed.

**Table 2:** Mean and SD of psychometric curve fit parameters.

Face type	*C_50_*	*R_max_*	*n*	*M*
Male neutral	0.602 (0.090)	0.926 (0.113)	9.036 (7.776)	0.061 (0.059)
Male sad	0.558 (0.087)	0.897 (0.133)	11.359 (15.528)	0.081 (0.084)
Female neutral	0.597 (0.108)	0.898 (0.120)	9.263 (7.496)	0.058 (0.071)
Female sad	0.612 (0.114)	0.905 (0.115)	9.176 (9.141)	0.066 (0.073)

#### Correlation Analysis

We hypothesized that the decision biases (decision threshold changes indexed by C_50_ differences) from negative facial expressions of male faces could be related to the body mass (BMI), depressive symptoms (BDI-II), ATOPs, or BAOPs that individual participants might have. To explore this possibility, we performed correlation analyses using C_50_ difference scores to index the decision biases due to sad facial expression (C_50_ of male neutral faces – C_50_ of male sad faces). As shown in **Figure 2D**, the perceptual decision biases of weight judgment (decreased perceptual threshold for male sad faces) showed a positive correlation with the BAOP scale, *r*(42) = 0.40, *p* = 0.007. To control an effect of outliers, we performed additional robust regression analysis. The result still showed a significant relation between the C_50_ difference and the BAOP scale, *b* = 28.35, *t*(42) = 2.47, *p* = 0.017. This finding suggests that the cognitive beliefs about obesity that participants had had an effect on how participants perceived the body weight of sad faces compared to neutral faces; specifically, participants who had stronger beliefs that obesity is not under the obese person’s own control tended to show a larger decrease in perceptual decision threshold. For the other measures, we could not observe any significant correlations.

## Discussion

Obesity is a rapidly growing public health concern, and more than two–thirds of adults in the United States are overweight or obese (Ng et al., 2014). Besides the health risks of obesity itself, “being fat” or fat-stigma is more than just a psychosocial stress. Indeed, overweight or obese individuals’ perception of being judged for their weight by others can negatively influence weight loss (Gudzune et al., 2014). Given the psychosocial implications of being judged as overweight or obese, it is important to better understand the perceptual decision-making processes underlying one’s judgment on another’s weight level.

The primary purpose of this study was to investigate how task-irrelevant emotional expressions influence judgments of body weight from faces. Additionally, this study sought to determine whether the relationship between emotional expression and weight judgment was modulated by participants’ explicit beliefs or attitudes toward obese people, affect, or their body masses. We first hypothesized that facial stimuli with sad affect would be judged as overweight more frequently than neutral affect facial stimuli. This hypothesis was supported in that an interaction was found between emotional condition and weight for male faces. Specifically, male sad faces were categorized as “fat” more frequently than male neutral faces at the 20, 40, 60, and 80% weight levels. Thus, it appears that task-irrelevant emotional expressions are capable of biasing subjective weight judgments of faces. Although the modulation of task-irrelevant emotional expressions on perceptual judgment of age, trustworthiness, and approachability has been previously reported (Willis et al., 2011; Voelkle et al., 2012), our study provides novel findings that emotional expression also systematically modulates weight judgments. Notably, the emotion-induced bias was observed only in male faces and not female faces. It also should be noted that there was no significant effect of stimulus gender on weight judgment for neutral faces. As shown in **Figure 2C**, the psychometric curves of male and female neutral faces very closely overlapped above each other, demonstrating that participants indeed employed identical perceptual decision threshold for weight judgments of each. The decision threshold decrease (i.e., a leftward shift of psychometric function) by sad facial expressions was specific to male faces, and this effect was not modulated by the sex of participants. The fact that the significant effect was found in the judgment of male faces but not female faces could be partly due to differences in societal expectations for the facial expressions among sexes. As stated before, sad emotion is typically linked with females (Kelly and Hutson-Comeaux, 1999). Thus, in our experiment, male faces with sad expressions might be harder to disregard while judging weight, whereas female faces with the same intensity level of emotional expressions might be relatively easier to disregard because they are less novel. Also, women tend to be judged more severely and with more negative psychosocial outcomes for their body weight than men, which is thought to be due to unequal value of the body between the two genders (Fikkan and Rothblum, 2012). This bias may play a role in the attentional focus of participants in this study, conceivably focusing more specifically on women’s weights while also being less focused on their emotion. Another albeit less likely explanation could be that the actual differences between men and women’s facial expressiveness (Kring and Gordon, 1998) that our study tried to experimentally control by using computer-generated faces somehow still played a role in modulating the perceived severity of the emotional expressions of different sexes.

It was also hypothesized that the weight decision bias of negative facial expressions may be related to the body mass, depressive symptoms, or attitudes or BAOPs that the individual participant holds. As mentioned previously, the BAOP scale, which measures beliefs about older persons, showed a significant positive correlation to emotion-induced perceptual decision biases of weight judgment. This suggests that participants who held stronger beliefs that obesity is not under the individual’s own control tended to show a larger decrease in perceptual decision threshold for male sad faces; that is, they tended to judge faces as overweight more sensitively than those with lower BAOP ratings. Thus, it might be plausible that participants who held the beliefs of external control of obesity would be more easily or sensitively influenced by other psychosocial factors such as the negative affect of face stimuli. We exploratively speculated that sad expression might serve as a relatively salient cue, particularly for the participants who had no blameful beliefs for the body weight of obese persons. However, our study itself could not directly provide evidence to support this claim, and further investigation will be needed to better understand the relationship between underlying cognitive beliefs toward obesity and weight judgment.

Instead of real faces that might have better external validity, our study choose to implement a computer-based face stimulus generation technique that has been widely used in recent emotion studies (Oosterhof and Todorov, 2009; Papesh and Goldinger, 2010; de Melo et al., 2014) and can provide better experimental controls that minimize potential confounding variables across different face sets. Critically, being able to morph the weight systematically (i.e., in equal 20% intervals) allowed us to control for any variance that would undoubtedly occur with weight changes across real photographs. However, based on previous research, it is not expected that the type of image (computer-generated or photographed faces) had a systematic impact on perceptual judgments.

We observed a significant interaction between sad emotional expressions and weight judgments in male faces, but many questions still remain. Our study had a relatively small sample size (44 college students), which may require further validation in a larger, more representative sample. Also, the lack of significant effect in female faces might be related to statistical power. It is still unclear whether other types of emotional expressions (e.g., happy, disgusted, angry, fearful) would systematically modulate weight judgments or not. Also, it is unknown whether psychiatric symptoms such as eating disorders and excessive body shape concerns modulate the emotion-induced biases of weight judgments. In our study, participants who had the stronger beliefs that genetic and environmental factors play critical roles in obesity showed larger emotion-induced perceptual decision changes. A recent study demonstrated that a short educational intervention for weight bias successfully reduced the BAOPs measured by the BAOP (Poustchi et al., 2013). Although our study itself cannot answer whether the emotion-induced perceptual decision bias is a stable trait or not, it would be informative to explore whether cognitive intervention can modify perceptual biases for weight decision.

Our findings shed new light on the impact that emotion, even when separated from the task itself, has on decisions about weight levels. They demonstrate the important, moderating role that emotion can play on subjective, perceptual judgment. This study is the first to examine whether sad affect modulates decision-making in the context of weight judgments. The impact that sad affect had on being perceived as overweight carries significant social implications for those who are overweight, although our study itself does not provide any explanation about a link between subjective weight judgment and subsequent social behaviors related to obesity stigma, which was beyond the scope of our experiment. This can be an important research topic in future studies. Nonetheless, this study not only better illuminates the role emotions play in basic perceptual judgments, but also gives further insight into how weight judgments, with their many and often severe social implications, can be biased by irrelevant, external factors such as emotion.
